# Decline in fish species diversity due to climatic and anthropogenic factors in Hakaluki Haor, an ecologically critical wetland in northeast Bangladesh

**DOI:** 10.1016/j.heliyon.2020.e05861

**Published:** 2021-01-06

**Authors:** Md. Saifullah Bin Aziz, Neaz A. Hasan, Md. Mostafizur Rahman Mondol, Md. Mehedi Alam, Mohammad Mahfujul Haque

**Affiliations:** a Department of Fisheries, University of Rajshahi, Rajshahi, Bangladesh; b Department of Aquaculture, Bangladesh Agricultural University, Mymensingh, Bangladesh

**Keywords:** Haor, Fish species diversity, Fishers, Principal component analysis, Climate change, Anthropogenic activity

## Abstract

This study evaluates changes in fish species diversity over time in Hakaluki Haor, an ecologically critical wetland in Bangladesh, and the factors affecting this diversity. Fish species diversity data were collected from fishers using participatory rural appraisal tools and the change in the fish species diversity was determined using Shannon-Wiener, Margalef's Richness and Pielou's Evenness indices. Principal component analysis (PCA) was conducted with a dataset of 150 fishers survey to characterize the major factors responsible for the reduction of fish species diversity. Out of 63 fish species, 83% of them were under the available category in 2008 which decreased to 51% in 2018. Fish species diversity indices for all 12 taxonomic orders in 2008 declined remarkably in 2018. The first PCA (climatic change) responsible for the reduced fish species diversity explained 24.05% of the variance and consisted of erratic rainfall (positive correlation coefficient 0.680), heavy rainfall (−0.544), temperature fluctuation (0.561), and *beel* siltation (0.503). The second PCA was anthropogenic activity, including the use of harmful fishing gear (0.702), application of urea to harvest fish (0.673), drying beels annually (0.531), and overfishing (0.513). Finally, the third PCA was loaded with the fishermen age (0.719), education (−0.767), and fishing experience (0.695) of the fishers. Deepening of *beels* could enhance dry season water availability and shelter the fish. Imposing fisheries regulations to reduce human activities is inevitable to sustain haor fisheries.

## 1 Introduction

**Figure 1:**
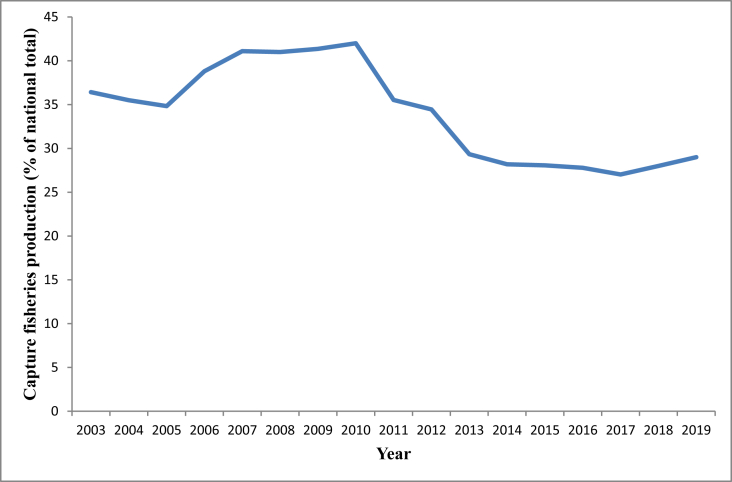
The trend of capture fisheries production (% of total national fish production) during the period 2003–2019 in Bangladesh (data source: Fisheries Resources Survey System - www.fisheries.gov.bd).

The improvement of the fisheries sector is an important agenda of the Bangladesh government for nutritional supply, income generation, and employment opportunities for an increasing population, along with much needed foreign exchange earnings. Globally, Bangladesh is a leading fish producing country, ranked third in inland fish production in 2018, after China and India [1]. Capture fisheries and aquaculture contribute significantly to rural diets and national food security by providing protein-rich food. Total fish production (2.1 million MT in 2003; 4.2 million MT in 2019) has increased over the years owing to increasing aquaculture production [2]; however, concomitantly the relative growth of capture fisheries is in decline (Figure 1). The production of capture fisheries depends heavily on different open water resources such as rivers, *beels*^1^, floodplains, and haors^2^. In Bangladesh, haors contribute approximately 10% of the total capture fisheries production [2], however this amount has reduced over time.

Among the total 373 reckoned haors, Hakaluki Haor is one of Asia's larger inland freshwater wetlands, with 80 inter-connected freshwater seasonal *beels* and is evenly distributed in the administrative area of the Fenchuganj and Golapganj Upazila from Sylhet district, and the Baralekha, Juri, and Kulaura Upazila from Moulvibazar district [3, 4]. Hakaluki Haor is designated as an ecologically critical area because a number of species from this ecosystem have already gone extinct [5]. Before extensive exploitation of this haor it had a plentiful supply of aquatic resources and provided shelter for brood fish in winter as it was covered by swamp forest. Fish species in Hakaluki Haor have drastically declined from 1993 to 2009 [6] due to unmanaged destruction of swamp forest as a result of limited conservation practices by the competitive authority, and the uncontrolled collection of wood for fuel and house construction, and the conversion of wetland areas to agriculture land and other purposes by the local people. Moreover, the sand deposition from upstream rivers has further dried up the swamp forest to a barren and consequently reduced the fish species diversity and production of fish by destroying important safe breeding habitats for fish and other aquatic animals. Furthermore, a failure to prevent illegal fishing has added further pressure to the fish stocks in Hakaluki Haor [7, 8]. An early study in 2005 reported the availability of about 100 fish species, one-third of which were endangered [7]. This number was reduced to 75 by 2009 [6] and also the diversity of each fish narrowed downed, illustrating a clear decline of fish species diversity in Hakaluki Haor.

Fish species diversity in the haors is the integral part of inland capture fisheries production that has been limited by a variety of factors, including habitat loss as a result of agricultural intensification, urbanization, environmental degradation and pollution, the over exploitation of resources, and climate change [9]. Fish production and diversity in natural waterbodies is extremely sensitive to climatic changes because feeding, migration, breeding, and other biological activities are affected by a complex set of water quality and weather parameters. In Bangladesh, one of most climatically vulnerable countries, haors have been reported as easily degraded wetlands and are facing increased pressures and threats from other factors including the overuse of resources by the local people [7].

In trying to understand these problems, most researchers have focused on revealing the changes in fish species diversity of Hakaluki Haor over time. Fish species diversity is a broad term which includes species richness (number of species in a defined area), species abundance (relative number of species) and phylogenetic diversity (relationships between different groups of species) of an ecosystem [10]. Moreover, fish species diversity encompasses the degree of nature's variety, including the number, frequency, and/or abundance of ecosystems, species, or genes in a given assemblage over a certain period of time [11, 12]. Assessing the species diversity of an aquatic ecosystem implies assessing the abundance of that assemblage. The temporal variation in species diversity, including abundance, richness, and evenness, are often observed within the assemblage. The available literature suggests that the depletion of aquatic species diversity is a common concern reported by several authors (for examples, see [13, 14, 15]); however, the factors affecting fish species diversity are not explicit in terms of climate change and/or anthropogenic activities. To date, there are no in-depth studies that show climate change and anthropogenic factors statistically associated with fish species diversity changes in haor ecosystems. Therefore, the present study aims to assess the changes of fish species diversity in Hakaluki Haor since 2008 and the factors responsible for these changes, focusing on two research questions: (i) is there a temporal decline in fish species diversity in Hakaluki Haor from 2008–2018 and (ii) if so, what are the reasons for the decline?

## 2 Materials and methods

### 2.1 Site selection

**Figure 2:**
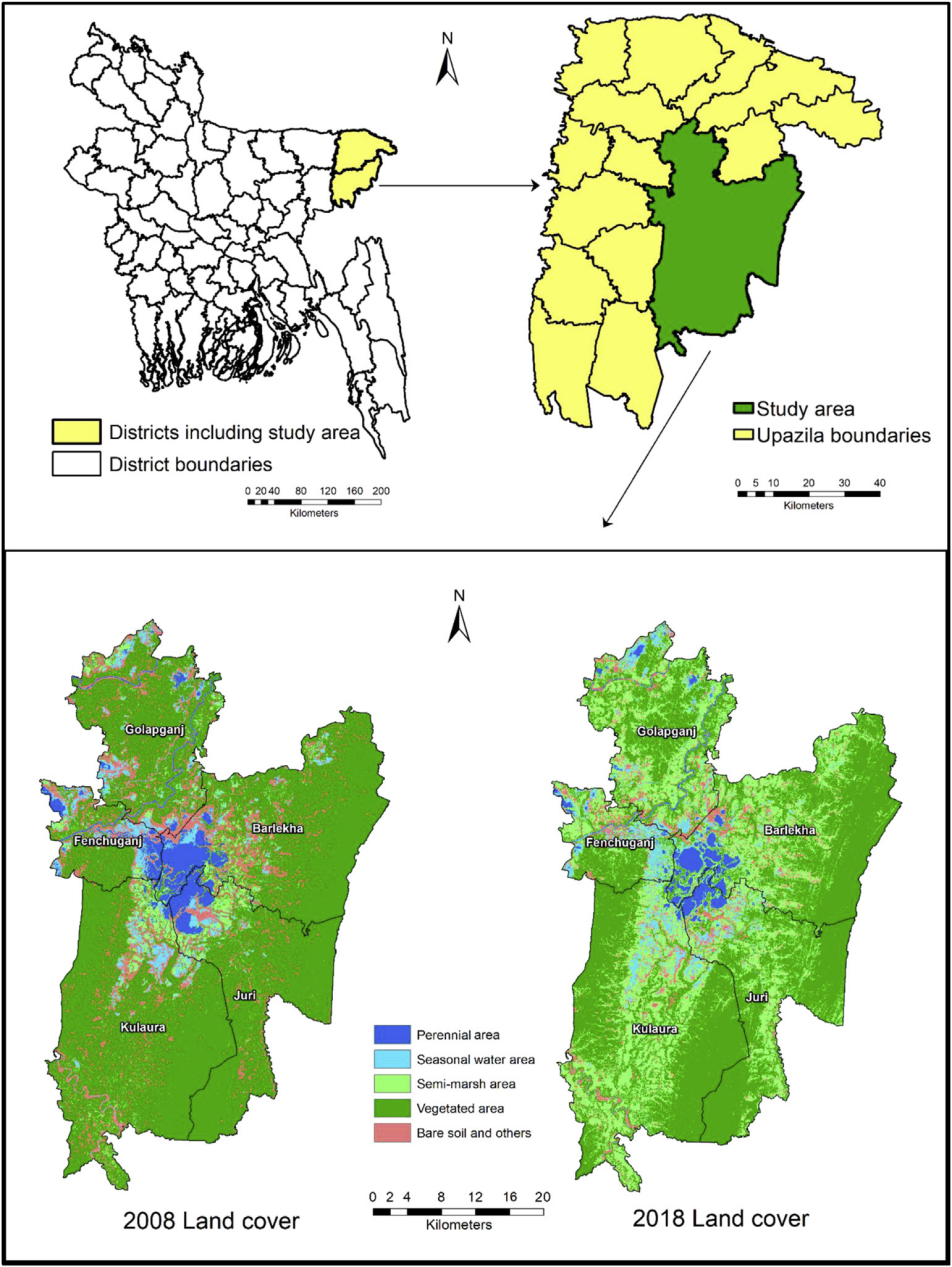
Spatial (GIS) map showing the study sites and changes in water area in the Hakaluki Haor from 2008 to 2018.

The study sites were selected from the five sub-districts encompassing Hakaluki Haor, the Barlekha, Juri, and Kulaura Upazila under the Moulvibazar district and the Fenchuganj and Golapganj Upazila under the Sylhet district of Bangladesh. These sites were selected because, in each of the sub-districts, an Upazila Fisheries Office under the Department of Fisheries (DoF) in the Ministry of Fisheries and Livestock, is tasked with regulating fishing and enhancing fish stocks with various interventions. These include the development of community-based fisheries management approaches (fisher-led, community-led, and women-led) allowing access to fish catch information from community-based management groups [16]. Moreover, fish species diversity is likely to vary between the five different sites owing to the availability of fishers in each location, their livelihoods strategies, and some other factors such as natural habitat destruction, conversion of waterbodies for other uses, and redirecting or obstructing the flowing water in the rivers and canals within haor. The geographic location of Hakaluki Haor is latitude 24°35′ N to 24°45′ N and longitude 92°00′ E to 92°08′ E where the comparative study sites in 2008 and 2018 are shown using spatial (GIS) map (Figure 2).

### 2.2 Selection of fish species diversity indices

We attempted to assess the fish species diversity of Hakaluki Haor by means of richness (number of different fish species), and evenness (dominated by a single species or with an equal number of individuals in each species category) of the fish assemblages based on the information of overall distribution and/or abundance (total number of individuals). The compound species diversity assessment index Shannon-Wiener index (*H*′) was chosen to represent the overall fish species diversity of Hakaluki Haor, in the form of richness. The Shannon-Wiener index [17] was calculated using the following formula:

(i) $$H^{\prime }=-\sum_{i=1}^{S}P_{i}\;\log\;2P_{i}$$

This can be rewritten as

(ii) $$H^{\prime }=\left\{3.3219\left(N\;\log\;N-\sum n_{i}-\log\;n_{i}\right)\right\}/N$$

Where *H′* = species diversity per individual, n_i_ = proportion of the samples belonging to the i^th^ species (Number of individuals of the i^th^ species), and N = total number of individuals in the collection.

The Shannon-Wiener index *H′* can express the richness of a fish population at a certain time point but cannot show temporal differences. For a better description of temporal changes in fish species richness in Hakaluki Haor we used the basic index, Margalef's Richness (*d*) [18]. The following formula (iii) was applied to determine the richness of fish in the haor [19].

(iii) d=(S−1)/logN

Where S = total number of species and N = total number of individuals in the sample.

To determine the distribution of individuals among the species present in the selected sites of Hakaluki Haor, an index of ‘evenness’ was chosen, which inherently represents the division of individuals across the species assemblage. The evenness was determined using formula (iv), the Pielou's Evenness index (*J′*) [20], which has a mathematical relationship with the Shannon-Wiener index *H′* [21].

(iv) $$J^{\prime }=H^{\prime }/log_{2}S\text{or }H^{\prime }/lnS$$

Where, *J'* = Evenness, *H′* = species diversity per individual, and S = total number of species.

### 2.3 Data gathering and analysis of fish species diversity

Fish species diversity data were collected in three different seasons [pre-monsoon (March–May), monsoon (June–August) and post-monsoon (September–November)] from fishers living in the five selected Upazilas using participatory rural appraisal tools [22]. Fifteen focus group discussions (FGDs) were conducted in five clusters, one in each of the five sites in each season, to develop a comprehensive list of the fishers (ranging from 100 to 150 individuals) in each individual study site. From these fisher lists, 30 fishers from each site (Barlekha, Juri, Kulaura, Fenchuganj, and Golapganj) (*N* = 150) were sampled using a random sampling technique.

To gather fish species diversity data between 2008–2018, FGDs were applied at the community level. In each cluster, during FGDs consisting of 10–15 fishers (including some key and experienced fishers), questions were asked to gather information on fish species diversity and a comprehensive list of the available fishes in haor was compiled. Commonly the fishers discard small and non-target fishes from their catch. Before the FGDs, fishers were asked not to discard any fish from their haul. One haul was equivalent to a catch per unit effort, meaning the total number of fish species caught by a group of fishers operating a seine net (ber jal^3^) for two and half hours (a standard duration of a single haul). Fishes from a single haul of all fishers were gathered in FGDs and individual fishers were asked to differentiate the fish species in different taxonomic categories. These groups of fish species diversity were crosschecked by the key and experienced fishers, and the data were recorded accordingly. Then the data of fish species diversity of all the individual fishers were compiled together in each FGD in a season (for details see Appendix A).

The fish species diversity data were collected by FGDs once a month between March and November 2018. The local names of the fish were crosschecked by key fishers in the FGDs who were born and lived in the same community and had extensive experience of fishing in Hakaluki Haor. In each FGD, after counting and recording the data for each fish species, fishers were asked to write the recall data for same species from the year 2008 when they were fishing. This recall data were further crosschecked by the same key and experienced fishers from each group.

### 2.4 Data analysis and assessment of fish species diversity indices

Data of fish species diversity, collated by site, species, season and temporal scale (2008–2018) were entered into an excel spreadsheet [23] and the fish species were arranged according to the taxonomic orders. These data were analyzed to obtain descriptive statistics of the changes of fish species availability between two years, as per the International Union for Conservation of Nature (IUCN) Red List. The composition data of seine net catch of all seasons were pooled together and analyzed to assess changes in fish species diversity over time. The Red List of Hakaluki Haor [24, 25] was applied in this study to represent the comparative availability of fish species under different taxonomic orders from 2008–2018. The descriptive statistics of the fish by site, year, taxonomic order, and species are presented in both tabular and graphical forms. The biodiversity indices such as Shannon's index, Margalef's Richness, and Pielou's Evenness were calculated using the data generated from FGDs conducted in the study sites for three seasons. The indices were calculated using the DIVERSE function available in PRIMER (version 7).

### 2.5 Data collection for analyzing factors associated with fish species diversity reduction

To understand the factors responsible for reducing fish species diversity, both structured and open-ended questions were included in a questionnaire relating to the socio-economic condition of the fishers and the climatic factors and anthropogenic activities associated with fish species diversity (For details see Appendix B). The questionnaire consisted of two sections, the first section focused on socioeconomic data about the fishers and the second section related to the factors affecting fish species diversity in the study sites. Before the survey, a draft questionnaire was prepared, pre-tested with two fishers in each site, and revised twice prior to finalization. The questionnaire survey was conducted between January and May 2019. Three competent enumerators conducted the survey of the sampled fishers (N = 150). Data collected from questionnaire interviews were entered into the MS Excel database for data management and statistical analysis. The collected data were analyzed using the multivariate statistical technique principal component analysis (PCA), as expressed by the following equation:

(v) $$PC_{i}=a_{1i}V_{1}+a_{2i}V_{2}+..........+a_{ni}V_{n}$$

Where *PC*_*i*_ is the principal component *i* and *a*_*ni*_ is (*n* = 1….*n*) the loading (correlation coefficient) of the original variables *V_n_* [26].

Changes to haor fish species diversity related to climate change and other anthropogenic activities are shaped by a large number of variables, which are difficult to interpret and relate to fish species diversity. The analysis of large datasets is increasingly common, and PCA is a widely used technique to reduce the number of variables and interpret these datasets [27] in a meaningful way. The objective of the PCA was to characterize the major factors responsible for the reduction of fish species diversity in Hakaluki Haor regardless of whether they were climate change - or anthropogenic activity-related. Statistical analyses were conducted using the statistical software SPSS 20 and Minitab 18. PCA extraction was carried out with a degree of subjectivity regarding the number of components. There are several ways of determining the number of components to be extracted, depending on a subjective ground framed by the researchers [28]. The common stopping rule to determine the number of components is to stop when the eigenvalue drops below 1 [29]. Therefore, eigenvalues >1 were employed to extract the number of components responsible for declining fish species diversity in Hakaluki Haor.

## 3 Results

### 3.1 Checklist of the fish species recorded

For the years 2008 and 2018 a total of 63 fish species were recorded from the FGDs, belonging to 12 taxonomic orders and 27 families. Table 1 shows the changes of catch composition consisting of 63 species between 2008 and 2018. It can be seen that catch composition of one haul by a seine net, in terms of the number of fish, decreased from 2008 to 2018. The most represented taxonomic orders were Cypriniformes, Perciformes and Siluriformes which made up over 80% of the total fish species in both the years. The most dominant species were *Mystus tengara, Eutropiichthys vacha, Chanda nama, Parambassis lala, Parambassis ranga, Gudusia chapra, Esomus danrica and Laubuka laubuca* in both years. On the other hand, the species with lowest representation in the catch were *Sperata aor, Rita rita, Channa striata, Monopterus cuchia, Macrognathus aculeatus, Notopterus notopterus, Chitala chitala, Labeo calbasu and Catla catla*. The most remarkable reductions in catch were recorded for the species of *Chanda nama, Gudusia chapra,* *Amblypharyngodon mola* and *Macrobrachium lamarrei*. A large decrease was reported in *Macrobrachium lamarrei*, a small prawn usually recognized as an indicator of healthy aquatic environments. Moreover, one species (*Chitala clitala*) under the taxonomic order of Osteoglossiformes had disappeared in 2018. Conversely, *Tenualosa ilisha* catch numbers increased slightly from 2008 to 2018. Interestingly, *Oreochromis mossambicus* was reported as a newly available species in 2018 (Table 1).

**Table 1:** Changes in fish catch composition by one haul of a seine net in Hakaluki Haor between 2008 and 2018.

Taxonomic Order	Family	Local name (in Bengali)	English name	Scientific name	Status as per IUCN (2015) in 2008	Status as per IUCN (2015) in 2018	Total catch (No of individual) of seine net in 2008	Total catch (No of individual) of seine net in 2018	Changes between the years (+/-)
Siluriformes	Bagridae	Bujuri tengra	Striped dwarf catfish	*Mystus tengara*	AV	AV	254	140	-
Siluriformes	Clariidae	Magur	Walking catfish	*Clarias batrachus*	AV	AV	80	6	-
Siluriformes	Heteropneustidae	Shing	Scorpion/Stinging catfish	*Heteropneustes fossilis*	AV	AV	110	15	-
Siluriformes	Schilbeidae	Batasi	Indian potasi	*Pseudeutropius atherinoides*	AV	AV	21	6	-
Siluriformes	Schilbeidae	Bacha	Batchwa vacha	*Eutropiichthys vacha*	AV	AV	150	92	-
Siluriformes	Siluridae	Boal	Freshwater shark/Boal	*Wallago attu*	NT	VU	17	5	-
Siluriformes	Siluridae	Madhu pabda	Pabda catfish	*Ompok pabda*	EN	CR	22	2	-
Siluriformes	Bagridae	Ayre	Long-whiskered catfish	*Sperata aor*	NT	VU	4	1	-
Siluriformes	Bagridae	Ritha	Rita	*Rita rita*	AV	NT	7	1	-
Siluriformes	Bagridae	Kabasi tengra	Gangetic mystus	*Mystus cavasius*	AV	NT	52	16	-
Siluriformes	Bagridae	Tengra	Striped dwarf catfish	*Mystus vittatus*	AV	VU	22	15	-
Perciformes	Channidae	Taki	Spotted snakehead	*Channa punctata*	AV	AV	40	5	-
Perciformes	Channidae	Shol	Striped snakehead	*Channa striata*	AV	AV	6	1	-
Perciformes	Centropomidae	Nama chanda	Elongate glass perchlet	*Chanda nama*	AV	AV	251	38	-
Perciformes	Anabantidae	Koi	Climbing perch	*Anabas testudineus*	AV	AV	79	13	-
Perciformes	Gobiidae	Bele	Gangetic tank goby	*Glossogobius giuris*	AV	AV	26	13	-
Perciformes	Channidae	Telo taki	Walking snakehead	*Channa orientalis*	AV	AV	25	23	-
Perciformes	Anabantidae	Khailsa	Striped gourami	*Colisa fasciata*	AV	AV	64	14	-
Perciformes	Anabantidae	Lal Khailsa	Dwarf gourami	*Colisa lalia*	AV	AV	36	14	-
Perciformes	Nandidae	Napit koi	Blue perch	*Badis badis*	AV	NT	24	7	-
Perciformes	Ambassidae	Lal Chanda	Highfin glassy perchlet	*Parambassis lala*	AV	VU	137	79	-
Perciformes	Centropomidae	Ranga chanda	Indian Glass Perch	*Parambassis ranga*	AV	VU	171	74	-
Perciformes	Nandidae	Meni	Gangetic leaffish	*Nandus nandus*	AV	NT	20	6	-
Perciformes	Channidae	Pipla shol	Barca snakehead	*Channa barca*	EN	CR	14	5	-
Perciformes	Cichlidae	Tilapia	Hawaiian perch	*Oreochromis mossambicus*	AB	AV	0	12	(+)
Synbranchiformes	Mastacembelidae	Guchi	Barred spiny eel	*Macrognathus pancalus*	AV	AV	37	12	-
Synbranchiformes	Synbranchidae	Kuchia	Swamp eel	*Monopterus cuchia*	NT	VU	12	1	-
Synbranchiformes	Mastacembelidae	Tara baim	Elephant trunk fish	*Macrognathus aculeatus*	AV	NT	15	5	-
Synbranchiformes	Mastacembelidae	Baim	Zig-zag eel	*Mastacembelus armatus*	AV	NT	14	1	-
Anabantiformes	Osphronemidae	Chuna Khalisha	Honey gourami	*Trichogaster chuna*	AV	AV	26	15	-
Beloniformes	Belonidae	Kakila	Asian needlefish	*Xenentodon cancila*	AV	AV	77	28	-
Tetraodontiformes	Tetraodontidae	Potka	Ocellated blowfish	*Tetraodon cutcutia*	AV	AV	15	8	-
Clupeiformes	Clupeidae	Ilish	Hilsa	*Tenualosa ilisha*	AV	AV	10	21	+
Clupeiformes	Clupeidae	Chapila	Indian river shad	*Gudusia chapra*	NT	VU	186	23	-
Cyprinodontiformes	Cyprinodontidae	Kanpona	Blue Panchax	*Aplocheilus panchax*	AV	AV	23	15	-
Osteoglossiformes	Notopteridae	Foli	Bronze featherback	*Notopterus notopterus*	NT	VU	20	4	-
Osteoglossiformes	Notopteridae	Chital	Clown knifefish	*Chitala chitala*	VU	EN	17	0	-
Cypriniformes	Cyprinidae	Darkina	Indian flying barb	*Esomus danrica*	AV	AV	173	75	-
Cypriniformes	Cyprinidae	Chap chela	Indian glass barb	*Laubuka laubuca*	AV	AV	127	45	-
Cypriniformes	Cyprinidae	Narkeli chela	Large razorbelly minnow	*Salmostoma bacaila*	AV	AV	62	16	-
Cypriniformes	Cyprinidae	Chala punti	Chola barb	*Puntius chola*	AV	AV	53	32	-
Cypriniformes	Cyprinidae	Teri puti	One spotted barb	*Puntius terio*	AV	AV	43	23	-
Cypriniformes	Cyprinidae	Jati-punti	Pool barb	*Puntius sophore*	AV	AV	57	12	-
Cypriniformes	Cyprinidae	Kanchan punti	Red/Rosy barb	*Puntius conchonius*	AV	AV	71	30	-
Cypriniformes	Cyprinidae	Mola punti	Glass-barb	*Puntius guganio*	AV	AV	53	12	-
Cypriniformes	Cyprinidae	Kalibaus	Kalbasu/Black rohu	*Labeo calbasu*	AV	AV	6	2	-
Cypriniformes	Cyprinidae	Rui	Rohu/Rui	*Labeo rohita*	AV	AV	87	13	-
Cypriniformes	Cyprinidae	Katal	Catla	*Catla catla*	AV	AV	7	2	-
Cypriniformes	Cobitidae	Gutum	Guntea loach	*Lepidocephalichthys guntea*	AV	AV	68	15	-
Cypriniformes	Balitoridae	Balichata	Mottled/Striped loach	*Acanthocobitis botia*	AV	AV	30	7	-
Cypriniformes	Cyprinidae	Phulo-chela	Finescale razorbelly minnow	*Salmostoma phulo*	AV	NT	57	6	-
Cypriniformes	Cyprinidae	Mola	Mola carplet	*Amblypharyngodon mola*	AV	NT	128	8	-
Cypriniformes	Cyprinidae	Dhela	Cotio/Dhela	*Osteobrama cotio*	AV	NT	39	3	-
Cypriniformes	Cyprinidae	Tit punti	Tic-tac-toe barb	*Puntius ticto*	NT	VU	41	10	-
Cypriniformes	Cyprinidae	Sarputi	Peninsular olive barb	*Puntius sarana*	AV	NT	16	3	-
Cypriniformes	Cyprinidae	Jelly puti	Golden barb	*Pethia gelius*	AV	NT	56	6	-
Cypriniformes	Cyprinidae	Reba carp	Reba carp	*Cirrhinus reba*	AV	NT	61	2	-
Cypriniformes	Cyprinidae	Mrigel	Mrigal	*Cirrhinus cirrhosus*	AV	NT	31	1	-
Cypriniformes	Cyprinidae	Bata	Bata labeo	*Labeo bata*	AV	NT	38	2	-
Cypriniformes	Cobitidae	Rani	Pakistani/Reticulate loach	*Botia lohachata*	VU	EN	15	3	-
Anguilliformes	Anguillidae	Banehara	Indian Longfin Eel	*Anguilla bengalensis*	AV	NT	41	5	-
Decapoda	Palaemonidae	Golda Chingri	Giant river prawn	*Macrobrachium rosenbergii*	AV	NT	45	2	-
Decapoda	Palaemonidae	Gura Chingri	Kuncho river prawn	*Macrobrachium lamarrei*	AV	NT	162	16	-

EN = Endangered, VU = Vulnerable, NT = Near Threatened, CR = Critically Endangered, AV = Available, AB = Absent.

+ → Increased, - → Decreased, (+) → Newly available.

### 3.2 Distribution of fish species on IUCN red list

**Figure 3:**
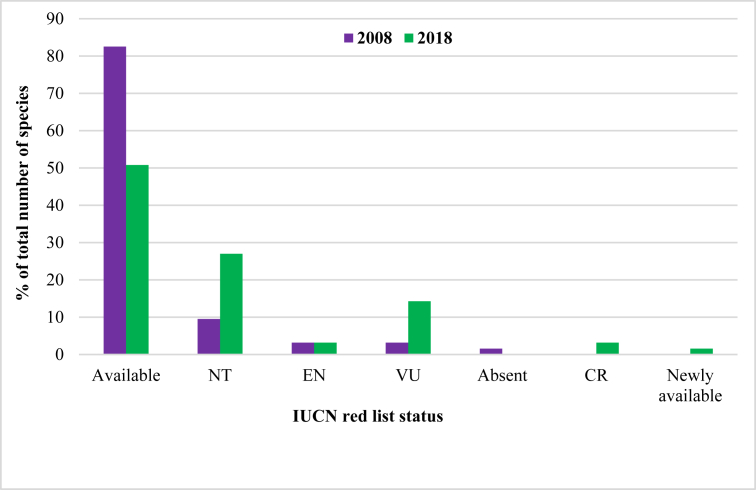
The status of fish species as per IUCN category in the year 2008 and 2018 (EN = Endangered, VU = Vulnerable, NT = Nearly Threatened, CR = Critically Endangered).

Figure 3 shows the changes in percent distribution of fish species in different categories of IUCN between the year 2008 and 2018. It is clear that in 2008, 83% of the fish species in Hakaluki Haor were in the available category (which are found readily) which decreased to about 51% in 2018. The distribution of nearly threatened and vulnerable species in 2008 were 9.5 and 3.17%, respectively which increased to 26.98 and 14.28% in 2018. About 3.17% of fish species were found endangered in both the years of study. In 2008, one species (1.58%) was unavailable but it became newly available species in 2018. In the year 2018, two species (3.17%) became critically endangered which were of the endangered category in 2008 (Figure 3).

### 3.3 Fish species diversity indices

Fish species diversity during different seasons in Hakaluki Haor changed over the years, as indicated by the calculated diversity indices (Table 2). The Shannon-Wiener index (*H′*) decreased from 2008 to 2018 in all the three seasons of sampling. The largest *H′* (3.812) was found in pre-monsoon/08 and smallest (3.418) in pre-monsoon/18 (Table 2). With the exception of Osteoglossiformes which showed an increase in the Shannon-Wiener index, a marked reduction in *H′* indices for the orders Siluriformes, Perciformes, Synbranchiformes, Anabantiformes, Beloniformes, Tetraodontiformes, Cypriniformes, Anguilliformes and Decapoda was observed between 2008 and 2018. However, during the same period of time, the reduction of *H′* indices for Clupeiformes and Cyprinodontiformes were comparatively low (Table 2). Compared to *H′*, the Margalef's Richness (*d*) slightly increased from 2008 to 2018 in all seasons and most taxonomic orders. However, the *d* of Osteoglossiformes and Clupeiformes declined slightly from 2008 to 2018. Pielou's Evenness (*J′*) denotes whether an individual species is evenly distributed in a particular geographical location. As with *H′*, the *J′* was consistently larger in 2008 for all seasons compared to that in 2018. *J′* was found to decline for all the taxonomic orders except Cyprinodontiformes from 2008 to 2018 (Table 2).

**Table 2:** Seasonal and order-wise fish species diversity indices found in Hakaluki Haor in 2008 and 2018.

Season	*H′* (log 2)		*d*		*J′*	
	2008	2018	2008	2018	2008	2018
Pre-monsoon	3.812	3.418	6.4325	6.536	0.6449	0.5773
Monsoon	3.762	3.499	6.3752	6.551	0.631	0.5576
Post-monsoon	3.763	3.436	6.4365	6.679	0.6312	0.5401
**Orders**	*H′* (log 2)		*d*		*J′*	

	2008	2018	2008	2018	2008	2018

Siluriformes	3.472	3.285	6.182	6.222	0.9284	0.8106
Perciformes	3.482	3.226	6.170	6.2127	0.9349	0.7736
Synbranchiformes	2.999	2.636	5.7226	5.7879	0.9459	0.717
Anabantiformes	2.477	2.199	5.2849	5.3344	0.9317	0.7565
Beloniformes	2.988	2.576	5.7487	5.8342	0.9388	0.679
Tetraodontiformes	2.481	2.264	5.2649	5.3558	0.9347	0.7976
Clupeiformes	3.002	2.867	5.7584	5.7061	0.9476	0.3671
Cyprinodontiformes	3.490	3.439	6.2047	6.3903	0.9398	0.9082
Osteoglossiformes	2.942	2.949	5.7482	5.7191	0.9098	0.4489
Cypriniformes	3.465	3.207	6.1644	6.1962	0.9243	0.7614
Anguilliformes	2.525	2.146	5.2701	5.3547	0.9624	0.723
Decapoda	3.451	3.053	6.1619	6.2536	0.9155	0.6643

*H′* - Shannon–Wiener index; *d* - Margalef's richness; *J′* - Pielou's Evenness.

### 3.4 Socioeconomic status of the fishers

For the surveyed fishers from all five sites, a set of socioeconomic data consisting of age, education, religion, gender, family status, occupational status, and annual income was collected. In all surveyed sites, the fishers were aged between 15 and 65, with the majority of these falling into the “31–50 year old” category. It was also observed that 50% of the fishers in all areas had finished primary school, while a negligible proportion had obtained a secondary level of education (Table 3). In terms of religion, there were mixed results between sites, with Barlekha and Golapganj dominated by Hindu fishers, and Muslim fishers dominating the other three sites. There was no participation by the female counterparts of the fishers reported, except some slight female participation in Barlekha. The surveyed fishers were classified according to fishing as their primary versus secondary occupation and, based on their annual income, they were categorized into ultra-poor, poor, moderately poor, lower middle class, and middle class. At every site, a notable number of fishers were below the marginal line, i.e. belonged to the poor group, except for Barlekha, where there was an even split between the poor and moderately poor groups.

**Table 3:** Socioeconomic characteristics of the fishers fishing in the selected sites of Hakaluki Haor (source: questionnaire survey).

Socio-economic characteristics		Number of fishers				
		Barlekha	Juri	Kulaura	Fenchuganj	Golapganj
Age	Young age (15–30)	7	9	8	9	9
	Middle age (31–50)	17	14	16	14	16
	Old age (51–65)	6	7	6	7	5
Education	Illiterate	12	10	11	11	10
	Primary level	14	15	17	16	18
	Secondary	4	5	2	3	2
Religion	Muslim	11	21	22	17	13
	Hindu	19	9	8	13	17
Gender	Male	26	30	30	30	30
	Female	4	0	0	0	0
Family status	Nuclear (≤4)	5	4	3	2	1
	Large (≥5)	25	26	27	28	29
Occupation	Primary (Fishermen)	27	28	25	26	22
	Secondary (Others)	3	2	5	4	8
Annual income	30,000–50,000 (Ultra-poor)	4	3	5	2	1
	50,000–80,000 (Poor)	8	12	15	18	16
	80,000–1,00000 (Moderately poor)	11	7	7	6	5
	1,00000–150,000 (Lower middle class)	4	5	3	3	6
	>150,000 (Middle class)	3	3	0	1	2

### 3.5 PCA of climate change, anthropogenic activities, and fish species diversity

**Figure 4:**
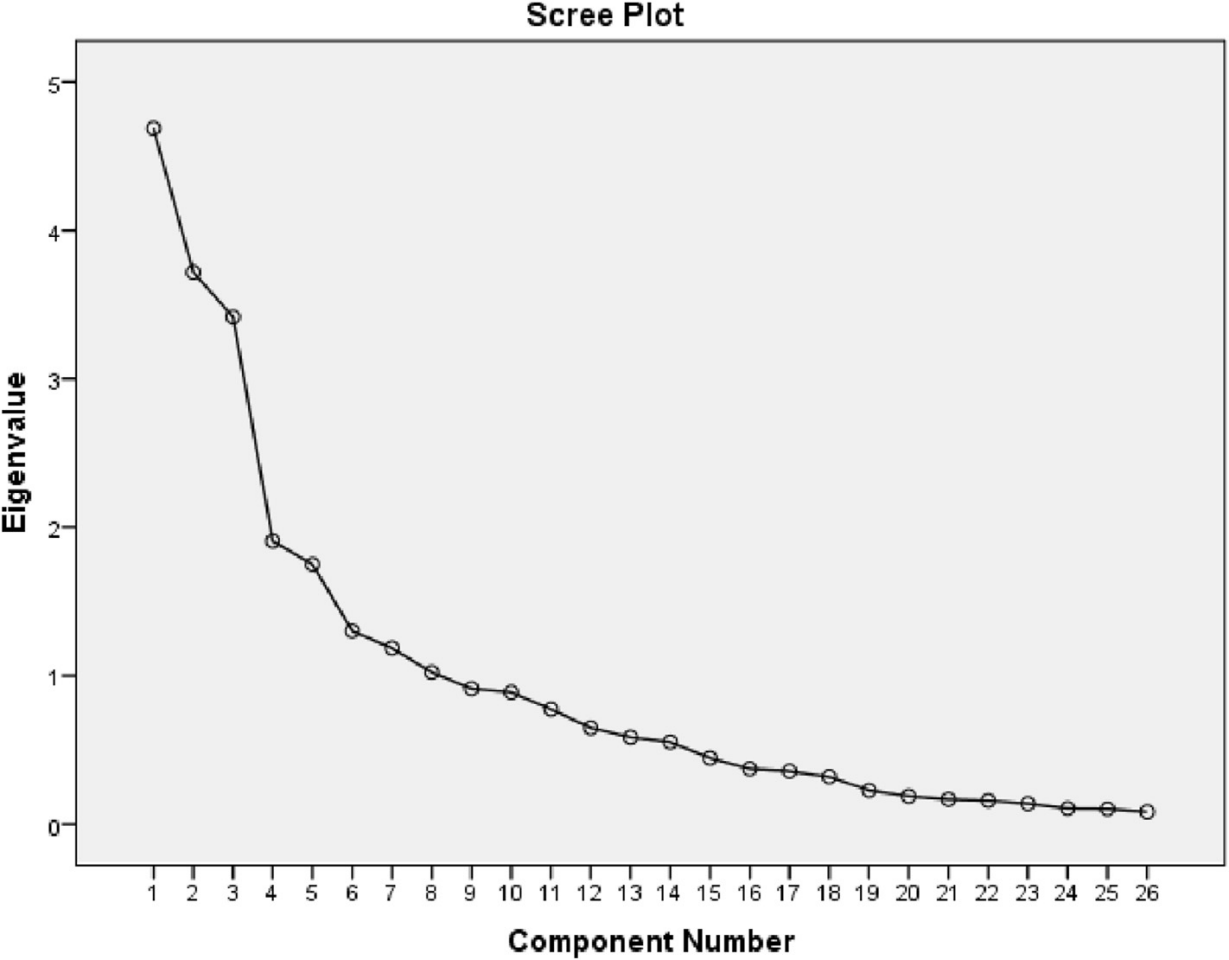
Scree plot (explained variance of each principal component) calculated for the factors.

**Figure 5:**
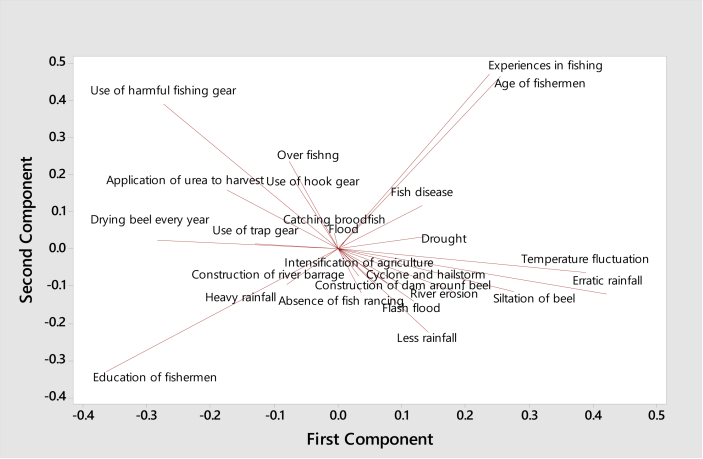
Loading plot corresponding to the top two principal components.

The scree plot of all the principal components is shown in Figure 4, of which the first three components have an Eigenvalue greater than 1, together explaining 49.30 % of the variance in the initial variables. As the scree plot clearly showed that the mentioned components could be retained, we chose to further analyze the first three components responsible for the declining fish species diversity in Hakaluki Haor. According to PCA extraction in SPSS, using a correlation matrix and standardized variables, the three components were extracted for overall factors, considering the noticeable step change in the scree plot. Moreover, in the loading plot, three distinct clusters have been developed with three different types of variables (Figure 5).

#### 3.5.1 Component 1: climatic change

PCA for the overall causes of the declining fish species diversity in Hakaluki Haor identified three main components. Component one was the climatic factors, which explained 24.05% of the variance (Table 4), and included erratic rainfall^4^, heavy rainfall, temperature fluctuation, and siltation in the *beels*. This extracted component describes the orientation of the climatic factors, either collectively or individually, with significant factor loadings. The erratic rainfall, temperature fluctuation, and siltation in the *beels* were loaded positively in this component. Individually, erratic rainfall, with a positive correlation coefficient (0.680), contributed the most to this component, followed by temperature fluctuation (0.561), and siltation in the *beels* (0.503), while heavy rainfall (−0.544) contributed negatively. It should be noted that all the variables associated with the climatic factors have relatively larger factor loadings with positive coefficients for the first component. In contrast, heavy rainfall had a negative factor loading with the lowest correlation coefficient (−0.544) on this component (Table 5).

**Table 4:** Eigenvalues of PCA of the dataset.

Principal component	Eigenvalues	% of variance	Cumulative % of variance
1	5.290	24.046	24.046
2	3.043	13.831	37.877
3	2.514	11.426	49.303
4	0.992	10.507	59.810
5	0.977	8.349	68.159
6	0.965	5.024	73.183
7	0.935	4.704	77.887
8	0.922	4.191	82.078
9	0.832	3.780	85.858
10	0.666	3.029	88.887
11	0.606	2.755	91.641
12	0.389	1.770	93.411
13	0.329	1.496	94.907
14	0.273	1.240	96.147
15	0.216	0.981	97.128
16	0.150	0.680	97.808
17	0.117	0.531	98.338
18	0.109	0.495	98.833
19	0.090	0.410	99.243
20	0.077	0.348	99.591
21	0.051	0.231	99.822
22	0.039	0.178	100.000

Extracted Method: Principal Component Analysis.

**Table 5:** Rotated component matrix and correlation coefficients of the variables with significant components affecting fish species diversity in Hakaluki Haor.

Rotated Component Matrix^a^			
	Component		
	1	2	3
**Erratic rainfall**	0.680		
**Heavy rainfall**	-0.544		
Less rainfall			
**Temperature fluctuation**	0.561		
Drought			
Flash flood			
Flood			
River erosion			
Cyclone and hailstorm			
**Siltation in *beel***	0.503		
Fish disease			
**Use of harmful gear**		0.702	
Use of trap gear			
Use of hook gear			
Construction of dam around *beel*			
**Application of urea to harvest fish**		0.673	
**Drying *beel* every year**		0.531	
Absence of fish ranching			
Construction of river barrage			
**Over fishing**		0.513	
Intensification of agriculture crops			
Catching broodfish			
**Age of fishermen**			0.719
**Education of fishermen**			-0.767
**Experiences in fishing**			0.695

Extraction Method: Principal Component Analysis.

Rotation Method: Varimax with Kaiser Normalization.

a Rotation converged in 13 iterations.

#### 3.5.2 Component 2: anthropogenic activities

This component was correlated positively with 13.83% of the variance (Table 4) and included destructive anthropogenic activities, i. e. the use of harmful gear, the application of urea fertilizer to harvest fish, drying the *beels* every year, and overfishing. Harmful gear showed the highest positive correlation coefficient. The use of harmful gear (0.702), applying urea (0.673), annual *beel* drying (0.531), and overfishing (0.513) (Table 5) built a clear image of the anthropogenic components responsible for the reduction of fish species diversity.

#### 3.5.3 Component 3: socioeconomic characteristics of the fishers

The age (0.719) and experience (0.695) characteristics of the fishers loaded significantly with positive correlation coefficients. In contrast, education (−0.767) loaded with a significantly negative correlation coefficient (Table 5).

## 4 Discussion

### 4.1 Status of fish abundance and fish species diversity

In the FGDs with the active participation of fishermen, we identified 63 species of fish and shellfish, belonging to 12 taxonomic orders in Hakaluki Haor in 2018. Using a similar FGD approach, Rahman et al. (2016) recorded 75 species belonging to 9 taxonomic orders from the Hakaluki Haor [30] and in 2015, two different studies identified 83 and 82 species belonging to 10 and 9 orders, respectively [8, 31]. However, the number of fish species per catch for several taxonomic orders decreased substantially between 2008 and 2018. The major decline in the numbers of individuals from different species was found in the Cypriniformes (mainly consisting of nutritionally important small indigenous fish species) and Decapoda. It is a well-established fact that small indigenous fish species such as *Chanda nama*, *Gudusia chapra,* *Amblypharyngodon mola* and *Macrobrachium lamarrei* are an easy and cheap source of vitamin A and calcium in the rural diet of the millions of poor people in Bangladesh [32]. In recent years, the increasing trend of small fish consumption by better off people has caused a very high market price of small indigenous fish [33]. For this reason, fishers enhanced their fishing pressure in the haor to harvest small fish which has resulted in the declining number in catches over the years. According to the IUCN red list category, the results of this study indicate that the fish species under the ‘available’ category declined from 83% to 51% between 2008 and 2018, and a large number of species fell into the nearly threatened (26.98%), vulnerable (14.28%), endangered (3.17%) and critically endangered (3.17%) categories. This change in distribution and changes in fish abundance over a 10-year period indicates fish species diversity in Hakaluki Haor is being negatively impacted, and there are possibilities of further damage if fishing practices remain the same.

Biodiversity can be assessed in various patterns, such as species diversity, genetic diversity, taxonomic diversity [34], phylogenetic diversity [35], chemical diversity, and functional diversity [36], where every pattern can be assessed by a group of indices. The main focus in this study was to assess temporal and seasonal fish species diversity, which is coherent with several diversity indices, like the number of species in an assemblage [37], richness [38], evenness [39], and rarity [40]. In this study, the variation of diversity between different orders and seasons was expressed by the *H′* index [41] which can be used to compare species diversity between two particular time frames. The *H′* was found to be higher for all three seasons in 2008 than those in 2018 which is an indication that the diversity of fish species in Hakaluki Haor was greater in 2008 than in 2018. The *H′* for all taxonomic orders of fish species were reduced to different extents over the years except the order Osteoglossiformes. This is possibly by virtue of an earlier massive freshwater flow into the haor from the Sonai river in Barlekha, the Juri river in Juri, and the Dhalai river in Kulaura which has reduced in recent years [42]. The *H′* of 2008 of this study is similar to a study in the south-east coast of India where the researchers illustrated seasonal impacts on the diversity of fish species with a maximum diversity in the pre-monsoon season [43]. Moreover, the high *H′* index (≥3.5) in 2008 for all seasons indicates a healthy and richly diverse area [44], which has declined for all seasons in 2018. The high *H′* index in 2008 also indicates that a large number of fish species thrived in the haor ecosystem by reproducing, feeding and sheltering themselves successfully [45]. Conversely, the low *H′* for all seasons of 2018 indicate the decline of haor diversity, possibly due to manmade and environmental stresses and/or the impacts of overfishing [46].

The Shannon-Wiener index *H′* deals with both the richness and abundance of an ecosystem while *d* is dedicated to assessing richness. In the case of richness, the difference between the two indices is that *d* is sensitive to sample size and that is applied to minimize the negative effects of the sampling process [47]. Apart from the taxonomic orders Clupeiformes and Osteoglossiformes, *d* for all orders in all seasons increased from 2008 to 2018. This is possibly due to the seasonal changes in species richness over the years that led to ecological differences between the orders. In terms of season for the observed years, the highest *d* was in post-monsoon 2018 where the lowest was in monsoon 2008. In Hakaluki Haor, parts of the DoF-administrated area (Barlekha, Juri, and Kulaura) lie on the Indian border [48]. Control over these transboundary areas is a possible cause for concern for the fishermen regarding the post-monsoon fishing as the Border Security Forces enhance their patrols. This security presence perhaps indirectly reduces the pressure of anthropogenic activities on fisheries resources, which results in the haor being protected and becoming a sanctuary for newborn fish in the post-monsoon period. This situation may be inverted in the monsoon when the border force relaxes their activities and the fishers take up their activities again. Globally, the UNEP World Conservation Monitoring Center has identified 3043 protected areas, among which 227 are adjoined to international boundaries [49]. In maximum security transboundary areas, species diversity is augmented over the years. For example, the Buenos Aires National Wildlife Refuge on the US–Mexico international boundary acts as free zone for the faunal and floral diversity, and protects the organisms from various anthropogenic destructive activities [50, 51]. Militarized and patrolling police activities in protected areas adjoining international boundaries also restrict transboundary human and invasive access through different degrees of legal, political, and law enforcement intensity, reducing effects of anthropogenic activities on natural ecosystem harmony [52, 53], thereby increasing the species diversity.

Evenness is the measure of relative diversity and this value becomes high when the entire area supports similar densities of species in a population, i.e. all species are distributed identically in a population [54]. Although this can also be assessed by the Simpson's Dominance [55], Berger–Parker [56], or Heip's [57] indices, the *J′* was chosen over the other indices for its mathematical relation with H′. The *J′* values of Hakaluki Haor decreased in all seasons of 2018 compared with 2008, and this decrease was significant for all taxonomic orders. All species of the orders had a homogenous distribution in 2008, which is clearly evident from almost similar *J′* indices of the taxonomic orders. However, the uneven *J′* indices of the taxonomic orders in 2018 indicate an irregular distribution of fish in Hakaluki Haor. One of the main reasons for this irregular distribution of fish species is the use of selective fishing gears by the fishermen in recent years [30]. Moreover, fishers catch more fish when the water level in the haor decreases, resulting in more species being caught since the monsoon is the breeding season of these freshwater fish [8]. This results in homogenous catches of particular orders of fish species i. e. low evenness indices. Such pattern of seasonal catches under specific orders has been reported in different waterbodies of Bangladesh in other studies [58, 59].

All the diversity indices determined in this study revealed that the fish species diversity of Hakaluki Haor decreased over time by seasons and taxonomic orders however, individual species were contributing evenly to the stock in recent years. Projects that aimed to limit human impacts on the hoar, such as the re-deepening of rivers to connect the associated waterways with the haors [60], the establishment of multitudinous sanctuaries by the Center for Natural Resource Studies (CNRS) and the DoF through their haor and Floodplain Resource Management projects [61], the promotion of a *beel* nursery program [61] and seasonal patrolling activities by the DoF to discourage haor fishers from using illegal fishing gears have all failed in their bid to improve the ecosystem. Species diversity in Hakaluki Haor can be restored by following community-based fisheries management such as the hilsa sanctuaries [62], developing plans to protect individual species in the haor ecosystem and forming a specialist group for working on haor diversity in Bangladesh like the IUCN Shark Specialist Group - SSG (www.iucnssg.org) or Global Marine Species Assessment - GMSA (http://sci.odu.edu/gmsa/) [63].

### 4.2 Factors affecting fish species diversity in Hakaluki Haor

#### 4.2.1 Climatic factors

In the first principal component, erratic rainfall emerged as a major climatic factor that has a positive relationship with reduced fish species diversity in Hakaluki Haor, thus affecting fish catch significantly. Generally, in haors the broodfish migration from the shelter of deeper water to the breeding ground starts from the end of February to first week of March. Erratic rainfall from the end of February to first week of March results in insufficient water supply in the rivers and *beel*s of haors; however, it quickly stimulates fish breeding. Some fish breed in the poor rainfall but owing to the lack of water larval development of fish does not occur [64]. Moreover, owing to the rainfall variability and lack of rainfall and water in the connected rivers and canals, the broodfish cannot reach the breeding grounds from haor in time [65]. Therefore, the climatic variability attributed to erratic rainfall affects the breeding activity and diversity of fish, compared to that a few decades ago [66].

Temperature fluctuation is another important factor that affects the physiological and ecological process that can translate into a decline in fish species diversity and distribution in a water body [67]. Temperature strongly controls all physiological processes particularly reproductive process such as gamete development, ovulation and spermiation, spawning, embryogenesis, and hatching to larval and juvenile development and survival which all have direct relationship with fish species diversity. Temperature fluctuations, a key climatic phenomenon, alter physico-chemical parameters of aquatic ecosystems and plankton productivity which translate into fish migration and distribution that has indirect and negative influence on abundances of fish [66].

Siltation in the *beels* showed a positive impact on the reduction of haor fisheries. It leads to the destruction of the natural habitat of the fish which has been evident in the spatial maps of Hakaluki Haor in 2008 and 2018 (Figure 2). The perennial water area, the most functional wetland of Hakaluki Haor in 2008, has remarkably been reduced in 2018 (Figure 2). This has resulted from continuous river erosion resulting from siltation and seasonal changes in the water regimes of *beels*, rivers and canals belonging to Hakaluki Haor. Although *beels* play an important role, the majority of *beels* have been silted up with 10–90% of their basins losing their resources [68]. Owing to this siltation, fish species diversity has been affected with a collapse of fish migration routes and destruction of 43% small *beels* which acted as feeding grounds [42, 69]. Moreover, owing to siltation at junction points between haors and rivers, the feeding grounds and migrations of fish larvae are hampered. Various aquatic habitats, i. e. *beel*s, rivers, and canals, show seasonal variability in their water regimes and the connectivity between the haor and the Kushiara river affect the fish species diversity. A functional river system makes a haor favorable for high fish production and, therefore, haors connected with flowing rivers tend to be very rich in fish production. The free discharge of water from the Kushiara river to Hakaluki Haor in the early monsoon promotes the migration of fish from the river to the haor. The varied depths of the haor basin provide habitats for young fish to grow, adults to mature, and broodfish to spawn in various suitable habitats [68]. The sediment concentrations, which typically range from 1,000 to 100,000 mg of sediment per liter of water can damage or kill fish [69]. Sediment intrusion from upper hill deforestation [70] and its deposition in *beels* was found to reduce light permeability and dissolved oxygen in the water, leading to lower survival of eggs, embryos and post-larvae [71] that leads toward to productivity reduction [72]. Increases in turbidity reduce light penetration in waterbodies [72, 73], which can lead to a reduction in primary productivity, as well as in the diversity of fish food organisms (secondary production), and finally in the species diversity of fish.

Compared to conditions in 1980, the proportion of degraded *beel*s has increased from 10% of the total *beel* area to more than 75%, causing a steady decline in fisheries resources [69]. These *beel*s are harbors for fish and other aquatic organisms necessary for fish production. The degradation of *beel*s has not only caused a decline in fish production, but also adversely impacted the livelihoods of local communities. Moreover, anthropogenic activities, such as intensive rice production, irrigation, and local dam construction, are affecting the ecological process, which in turn is hampering the production of resources and reducing the economic value of *beel*. As a result of this degradation, 75 (out of 75) of the large *beel*s and only 36 (out of 63) of the small *beel*s in Hakaluki Haor contain resources, as listed by the Ministry of Land [69], and the remaining *beels* are not providing the expected level of fish supply.

#### 4.2.2 Anthropogenic factors

There are several anthropogenic factors that affect fish species diversity directly and indirectly. Directly the use of harmful fishing gear is the most important anthropogenic factor that has led to the decline of fish species diversity in the haor. Synthetic gill nets (current jal^5^) and seine nets (bher jal^6^) are the most harmful fishing gear used in Hakaluki Haor over the years [30]. Fish eggs and fry can easily be captured by fishers using these fishing gears. In the FGDs, fishers reported setting traps for small fish along their migration route, thereby easily capturing fish fry and eggs. Concomitantly, the broodfish swim close to the bank of the haor at night, and are caught using harpoons (dragging gear) by the fishers. The use of those destructive fishing gears and some other methods (for example, fishing by de-watering, use of chemicals), makes fish and other aquatic organisms vulnerable to exploitation and has led to the regulatory authority (DoF) deeming these as illegal fishing techniques [30]. However, fishers in haor regions continue to use harmful fishing gear and techniques in the absence of effective monitoring by the regulatory authority. This has led to a decline in fish species diversity [74]. For the conservation of fish species and their habitats, DoF has also placed restrictions on catching brood fish and juveniles from natural sources. However, owing to poor regulatory capacity, no visible impacts on the conservation of fish species were observed [69]. Indirectly, anthropogenic climate change has impacted the haors due to altered patterns in water temperature and extreme rainfall events. These have translated into various changes in the aquatic systems and impact on fish species diversity as discussed above [75].

Overfishing is another anthropogenic activity identified from PCA which reduced fish species diversity in Hakaluki Haor. A survey conducted in 1993 counted 107 fish species; however the number of species found in 2009 was 75 [6]. About 91% of the fishers thought that over exploitation of resources is occurring owing to an increased number of fishers and their dependency on haor resources [76]. Around 190,000 people living the surrounding areas of Hakaluki Haor utilize the resources year-round causing a decline an wetlands [76]. Two main sources of livelihood for the people in Hakaluki Haor are capture fisheries and agriculture. This decline in wetlands has resulted in more than 40% of the freshwater fish species being classed as threatened with national extinction [77]. With a negative impact on fish species diversity, Hakaluki Haor is in danger of losing nearly 32 fish species out of 107 because of overfishing, which is a serious threat to fish stocks in the haor [30].

#### 4.2.3 Socioeconomic factors

Socio-economically, the fishers in haor areas tend to be very poor and are extremely dependent on fishing, which has direct negative impacts on fish species biodiversity (Table 4). Moreover, the better-off fishers are also detrimental to the fish species diversity in haors as they can invest more money in buying boats and fishing gear [78].

Age was the most vital factor that affected the behavior of the fishers in the reduction of fish species diversity in the haors. From FGDs, it was evident that the older fishers had a tendency to catch fish as per their traditional practices, without following the regulations imposed by the DoF in recent years. Moreover, the older fishers are often illiterate and tend to have poor knowledge of fishing regulations. In contrast, the younger literate fishers have a relatively better understanding of the fish breeding seasons and government regulations. Poor education may indirectly affect indiscriminate fishing tendency of fishers which results in reduction of fish species diversity in the haor. Educated fishers tend to follow fishing regulations; however, the less educated fishers do not. Experienced fishers catch more fish than less experienced ones. The indiscriminate fishing by poor, illiterate and experienced fishers will have serious consequences in terms of a reduction of fish species diversity in the haors.

## 5 Conclusion

A comprehensive study was undertaken to examine the changes of fish species diversity over 10 years from 2008–2018. A total of 69 species of fish and shellfish from 12 taxonomic orders were recorded. The Shannon-Wiener index showed that the diversity of fish in the haor reduced from 2008 to 2018. Margalef's Richness index was higher in the monsoon/2008 season than in the monsoon/2018 season. The highest Pielou's Evenness was found in the pre-monsoon/2018 season. The PCA results highlight three components, the first identifying the role of erratic rainfall, temperature fluctuation, and siltation in the *beels* for the reduction of fish species diversity. The second component showed positive correlations between destructive anthropogenic activities, including the use of harmful fishing gear, applying urea fertilizers for fishing, annual drying of *beels*, and overfishing, with a reduction of fish in the haors. This study suggests that fish species diversity is declining in Hakaluki Haor owing to climate change, anthropogenic activities and socioeconomic condition of fishers.

To conserve the haor ecosystem, the government is trying to promote co-management to attain increased fish production, enhanced biodiversity, and sustainable livelihoods for fishers. To retain and enhance these activities, the Community-Based Adaptation in the Ecologically Critical Haors through Biodiversity Conservation and Social Protection Project was taken by the Department of Environment. The DoF has adopted a number of strategies, such as i) sustainable fishing; ii) the conversion of part or the whole of a haor into a sanctuary; iii) banning the use of current nets and other harmful fishing gear; iv) banning the drying of waterbodies; v) the deepening and renovation of waterbodies in haors; and vi) diversifying alternative income generating activities for fishers. However, these kinds of activities are not carried out consistently in coordination with other agencies. Therefore, consistent and coordinated mitigation measures should be taken by government and non-government organizations for the conservation of fish species diversity in Hakaluki Haor.

## Declarations

### Author contribution statement

Md. Saifullah Bin Aziz: Conceived and designed the experiments; Performed the experiments; Wrote the paper.

Neaz A. Hasan, Md. Mehedi Alam: Analyzed and interpreted the data; Wrote the paper.

Md. Mostafizur Rahman Mondol, Mohammad Mahfujul Haque: Conceived and designed the experiments; Contributed materials, analysis tools or data; Wrote the paper.

### Funding statement

This research did not receive any specific grant from funding agencies in the public, commercial, or not-for-profit sectors.

### Data availability statement

Data associated with this study has been deposited at Mendeley under the accession number https://doi.org/10.17632/v9z8fckpfh.1.

### Declaration of interests statement

The authors declare no conflict of interest.

### Additional information

No additional information is available for this paper.

## Appendix A Supplementary data

AppendixA AppendixA

AppendixB AppendixB

## Footnotes

1 Perennial low-lying depression on a floodplain, sometimes drying up in the dry season and smaller than a haor.

2 Saucer-shaped, low-lying depression on a floodplain or river swamp, part of which dries up during the dry season.

3 A ber jal is a rectangular net having large wall of netting and has two border lines. This gear is effective in catching both small and large species of fish in haors and other open waterbodies.

4 Erratic rainfall indicates both increases and decreases in precipitation that occur at unexpected times or follow an irregular pattern.

5 A current jal is a string of single, double, or triple netted walls, positioned vertically near the surface, midwater, or on the bottom, in which fish or their gills become entangled or enmeshed.

6 A bher jal is a very long net, with or without a bag in the centre, which is set either from the shore or from a boat to surround a certain area. It is operated with two (long) ropes fixed to its ends, one for hauling and the other for herding the fish.
