# Supplementary material for: Decline in fish species diversity due to climatic and anthropogenic factors in Hakaluki Haor, an ecologically critical wetland in northeast Bangladesh
Source: Heliyon. 2021 Jan 6;7(1):e05861. doi: 10.1016/j.heliyon.2020.e05861 (PMC7855352; doi:10.1016/j.heliyon.2020.e05861)
Supplement: AppendixA [file mmc1.docx]

**Appendix A: Field notes for the fishers participants of the Focus Group Discussion to record the data of fish species diversity**

| Focus group data recording pro-forma | | |
| --- | --- | --- |
| Study title: |  | |
| Location: |  | |
| Date: |  | |
| Moderator name: |  | |
| Pro-forma completed by: |  | |
| Group details: |  | |
| No. of participants: |  | |
| Start time: |  | |
| End time: |  | |
| Participants demographic: | | |
| Name | Gender | Age |
|  |  |  |
|  |  |  |
|  |  |  |
|  |  |  |

**Repertory grid for fish species diversity assessment**

Here all the selected fishers were communicated previously before arranging the focus group discussion. On focus group discussion day, the participatory fishers were gathered in a place with their harvest fish. Then they were asked to categorize the fish in their catch according to the species category. In every focus group of all sites, some key experienced fishers were included to cross-check the fish groups and finally the data was recorded in the following table.

| Scientific name of the species | Local name of the species | Catch from one haul by group-1 | Catch from one haul by group-2 | Catch from one haul by group-3 | Catch from one haul by group-4 | Catch from one haul by group-5 | Catch from one haul by group-6 | Catch from one haul by group-7 | Catch from one haul by group-8 | Catch from one haul by group-9 | Catch from one haul by group-10 | Catch from one haul by group-11 | Catch from one haul by group-12 | Catch from one haul by group-13 | Catch from one haul by group-14 | Assessment note from experienced fishers |
| --- | --- | --- | --- | --- | --- | --- | --- | --- | --- | --- | --- | --- | --- | --- | --- | --- |
|  |  |  |  |  |  |  |  |  |  |  |  |  |  |  |  |  |
|  |  |  |  |  |  |  |  |  |  |  |  |  |  |  |  |  |
|  |  |  |  |  |  |  |  |  |  |  |  |  |  |  |  |  |

**Repertory grid for recall fish species diversity assessment**

After grouping and recording the fish catch by species the participating fishers were asked to provide recall data for each species based on experience of their previous catch. This data was again cross-checked by the key experienced fishers present in focus group discussion.

| Scientific name of the species | Local name of the species | Catch from one haul by group-1 | Catch from one haul by group-2 | Catch from one haul by group-3 | Catch from one haul by group-4 | Catch from one haul by group-5 | Catch from one haul by group-6 | Catch from one haul by group-7 | Catch from one haul by group-8 | Catch from one haul by group-9 | Catch from one haul by group-10 | Catch from one haul by group-11 | Catch from one haul by group-12 | Catch from one haul by group-13 | Catch from one haul by group-14 | Assessment note from experienced fishers |
| --- | --- | --- | --- | --- | --- | --- | --- | --- | --- | --- | --- | --- | --- | --- | --- | --- |
|  |  |  |  |  |  |  |  |  |  |  |  |  |  |  |  |  |
|  |  |  |  |  |  |  |  |  |  |  |  |  |  |  |  |  |
|  |  |  |  |  |  |  |  |  |  |  |  |  |  |  |  |  |
