# Supplementary material for: Decline in fish species diversity due to climatic and anthropogenic factors in Hakaluki Haor, an ecologically critical wetland in northeast Bangladesh
Source: Heliyon. 2021 Jan 6;7(1):e05861. doi: 10.1016/j.heliyon.2020.e05861 (PMC7855352; doi:10.1016/j.heliyon.2020.e05861)
Supplement: AppendixB [file mmc2.docx]

# Appendix B: Questionnaire for fishers survey on climate change in Hakaluki Haor

**Fishers’ ID:**

| Interviewee’s name: |  | | | | |
| --- | --- | --- | --- | --- | --- |
| Interviewee’s age: | |  | | | |
| Interviewee’s gender: | | 1=Male 2=Female | | | |
| Level of education  (Number of schooling year): | |  | | | |
| Interviewee’s address: | |  | | | |
| Contact details: | |  | | Mob. No: |  |
| What is your role in the family? | | |  | | |
| No. of years in this area? /Years | | | | | |
| No. of years on fishing? /Years | | | | | |
| Interview date: | | | | | |
| GPS: | | | | | |

| Primary occupation: | |  | | | | | | |
| --- | --- | --- | --- | --- | --- | --- | --- | --- |
| Secondary occupation: | | |  | | | | | |
| Religion: | | | 1=Muslim 2=Hindu | | | | | |
| House hold member: | | |  | | | | | |
| Number of Male members | | | | |  | | | |
| Number of female members | | | |  | | | | |
| Land ownership | Own | | |  | |  | Lease |  |

| Main income in amount (BDT) |  |
| --- | --- |
| Secondary income in amount (BDT) |  |
| Is there any change of the main and secondary occupation? |  |

**Issues related to climate change**

| Do they believe in climate change? (Yes/Not) |  |
| --- | --- |
| Seasonal variation occurred (Yes/No) |  |
| Seasonal variation at past and present |  |
| Rain pattern change (Yes/No) |  |
| Rain pattern change at past and present |  |
| Temperature extreme (Yes/No) |  |
| Temperature extreme at past and present |  |
| Natural calamity (Yes/No) |  |
| Natural calamity at past and present |  |
| River level rise (Yes/No) |  |
| River level rise at past and present |  |
| Flash flood occurred year |  |
| Flash flood occurred at past and present |  |

**Issues related to fish species diversity**

| Availability of fish species 10 years back |  |
| --- | --- |
| Which Species of fish are now extinct |  |
| How long of these fish species are extinct? |  |
| Which Species of fish are now less |  |
| Why they are extinct or less? |  |

**Major cause of fish reduction in Hakaluki Haor**

| Rank | Scale |
| --- | --- |
| 4 | Highly affected |
| 3 | Moderately affected |
| 2 | Less affected |
| 1 | Not affected |

1. Weather variables
2. Temperature fluctuation

| 1 | 2 | 3 | 4 |
| --- | --- | --- | --- |
|  |  |  |  |

1. Erratic rainfall

| 1 | 2 | 3 | 4 |
| --- | --- | --- | --- |
|  |  |  |  |

1. Heavy rainfall

| 1 | 2 | 3 | 4 |
| --- | --- | --- | --- |
|  |  |  |  |

1. Less rainfall

| 1 | 2 | 3 | 4 |
| --- | --- | --- | --- |
|  |  |  |  |

1. Drought

| 1 | 2 | 3 | 4 |
| --- | --- | --- | --- |
|  |  |  |  |

1. Flash flood

| 1 | 2 | 3 | 4 |
| --- | --- | --- | --- |
|  |  |  |  |

1. Flood

| 1 | 2 | 3 | 4 |
| --- | --- | --- | --- |
|  |  |  |  |

1. River erosion

| 1 | 2 | 3 | 4 |
| --- | --- | --- | --- |
|  |  |  |  |

1. Natural disaster (Cyclone and hailstorm)

| 1 | 2 | 3 | 4 |
| --- | --- | --- | --- |
|  |  |  |  |

1. Siltation in *beel*

| 1 | 2 | 3 | 4 |
| --- | --- | --- | --- |
|  |  |  |  |

1. Fish disease

| 1 | 2 | 3 | 4 |
| --- | --- | --- | --- |
|  |  |  |  |

1. Manmade cause
2. Use of harmful gear

| 1 | 2 | 3 | 4 |
| --- | --- | --- | --- |
|  |  |  |  |

1. Use of trap gear

| 1 | 2 | 3 | 4 |
| --- | --- | --- | --- |
|  |  |  |  |

1. Use of hook gear

| 1 | 2 | 3 | 4 |
| --- | --- | --- | --- |
|  |  |  |  |

1. Construction of dam around *beel*

| 1 | 2 | 3 | 4 |
| --- | --- | --- | --- |
|  |  |  |  |

1. Application of urea to harvest fish

| 1 | 2 | 3 | 4 |
| --- | --- | --- | --- |
|  |  |  |  |

1. Drying *beel* every year

| 1 | 2 | 3 | 4 |
| --- | --- | --- | --- |
|  |  |  |  |

1. Absence of fish ranching

| 1 | 2 | 3 | 4 |
| --- | --- | --- | --- |
|  |  |  |  |

1. Construction of river barrage

| 1 | 2 | 3 | 4 |
| --- | --- | --- | --- |
|  |  |  |  |

1. Over fishing

| 1 | 2 | 3 | 4 |
| --- | --- | --- | --- |
|  |  |  |  |

1. Intensification of agriculture crops

| 1 | 2 | 3 | 4 |
| --- | --- | --- | --- |
|  |  |  |  |

1. Catching broodfish

| 1 | 2 | 3 | 4 |
| --- | --- | --- | --- |
|  |  |  |  |

1. Scocio-economic cause
2. Age of fishermen

| 1 | 2 | 3 | 4 |
| --- | --- | --- | --- |
|  |  |  |  |

1. Education of fishermen

| 1 | 2 | 3 | 4 |
| --- | --- | --- | --- |
|  |  |  |  |

1. Experiences in fishing

| 1 | 2 | 3 | 4 |
| --- | --- | --- | --- |
|  |  |  |  |
